# mSphere of Influence: Positive Research Culture Enables Excellence and Innovation

**DOI:** 10.1128/mSphere.00948-19

**Published:** 2020-01-15

**Authors:** Elizabeth R. Ballou

**Affiliations:** a Institute of Microbiology and Infection, School of Biosciences, University of Birmingham, Birmingham, United Kingdom

**Keywords:** mSphere of Influence, medical mycology, microbiology

## Abstract

Elizabeth Ballou works in the field of medical mycology. In this mSphere of Influence article, she reflects on how two papers by Okagaki et al. (PLoS Pathog 6:e1000953, 2010, https://doi.org/10.1371/journal.ppat.1000953) and Zaragoza et al. (PLoS Pathog 6:e1000945, 2010, https://doi.org/10.1371/journal.ppat.1000945) made an impact on her career by demonstrating an alternative to destructive publication practices.

## Abstract (precis)

Elizabeth Ballou works in the field of medical mycology.

## COMMENTARY

Too often, those credited with scientific breakthroughs also enable poor research culture. Examples of gatekeepers, bad actors, and bullies are easy to find throughout the scientific literature, with stories passed on to the next generation through whispered conversations over coffee or in the pub. But how we go about our work, how we interact with our colleagues, and how we train our students have lasting impacts on the course of research. A positive research culture creates space and opportunity for new perspectives, risk, and creativity. These are the drivers of discovery and innovation. Jeremy Farrar, Director of the Wellcome Trust, recently called on each of us to make positive research culture a shared goal (1). The focus of Wellcome reform efforts is on harassment and bullying, and achieving diversity and inclusion goals: systemic challenges that are the domain of universities, funders, and hiring managers. However, we as researchers, collaborators, reviewers, and editors likewise have a role to play in ensuring that the most creative researchers are represented and add their influence to our shared human endeavor.

In my short career, I have twice benefited from the leadership of mycologists in this sphere. In 2009, at the Marine Biology Laboratory’s course on Medical Mycology, I met a fellow graduate student, Laura Okagaki, who had an exciting story to tell about an unusual observation that had been long overlooked as an artifact in the literature (2–4). Okagaki was working in the lab of Kirsten Nielsen, then a new principal investigator (PI) at the University of Minnesota, and the two were about to demonstrate the existence of Cryptococcus neoformans titan cells as an *in vivo* phenomenon (5). Okagaki was anxious about discussing the story before it was published, but we developed a friendship over our shared interest in C. neoformans morphogenesis, and I promised to send her mutants I had generated that we suspected might influence the process (6). Shortly afterward, it became clear that a similar story was being developed in Arturo Casadevall’s group, led by postdoc Oscar Zaragoza (7). In some fields, this turn of events has signaled the inevitable end of a career, as high-risk projects that have consumed resources and precious postdoc and graduate student time turn into “me too” papers replicating scooped work.

It took true leadership and a long view of the power of positive research culture to challenge this narrative. Rather than competing to be first to publish, Nielsen and Casadevall worked together and published the reports back to back in *PLoS Pathogens* in 2010 (5, 7). The positive impact of this on the scientists directly involved is obvious: both labs were able to publish their work in a well-respected journal and received fair and independent reviews unbiased by the artificial shadow of “novelty.” The two papers are distinct in their approaches, and their findings reinforce rather than directly replicate each other, enabling increased confidence in the importance of the phenotype to C. neoformans pathogenesis from the wider community. This approach also ensured that both Nielsen and Zaragoza could go on to publish important subsequent work which has been the foundation of a whole field of research with implications for patient outcomes (8, 9).

What may surprise readers is that the wider implications of this leadership are still being felt today. In 2017, I launched my lab at the University of Birmingham, United Kingdom, with the goal of studying mechanisms driving titanization. Despite much effort on the part of Nielsen and Zaragoza’s groups, neither had been able to replicate the phenotype *in vitro*, making mechanistic studies challenging. However, an undergraduate in my lab, Tom Drake, had observed that titan cells can be induced *in vitro* using a simple culture technique, which we subsequently demonstrated is the result of cross-kingdom signaling between fungi and bacteria (10). The discovery was thrilling and opened up rapid access to research avenues that had previously been inaccessible. But my lab at the time consisted of two 6-week summer students and Drake, who was having such fun he just kept turning up every day after graduation. So I reached out to Nielsen to ask if she would be willing to collaborate. She told me two other labs, led by Zaragoza and another new PI, Alexandre Alanio, had already contacted her with similar stories. She was collaborating with Alanio, and they expected to submit the paper within the year.

It was my first time leading a research project and a huge risk to focus my small lab’s efforts and limited funding on an extremely competitive area, particularly now that they knew I was working on a paper. Fortunately, Nielsen immediately encouraged me to contact the editors at *PLoS Pathogens* to ask them to again consider the works as complementary reports. After reaching out to Alanio and Zaragoza and the editor and staff at *PLoS Pathogens*, we were able to coordinate our papers, and all three were published back to back to back in 2018 (10–12). Again, our papers are complementary rather than directly replicative, and our overlapping findings further reinforce the deep signaling events regulating this important morphogenetic switch. The example first established by Nielsen and Casadevall benefited not only the PIs on the three papers, but also our teams, particularly graduate students Hommel and Trevijano-Contador, who led the work in the Alanio and Zaragoza labs. We all continue to work in this space, contributing to a broader understanding of the many interacting signals that drive titanization (13). It has also served as an example for others facing similar competitive choices in our field (14–17).

The practice of scooping and the artificial value we place on being first to publish reveal the lie that science is a meritocracy, where the best research rises to the top. Rather, it is the best resourced that can compete and dictate what work is recognized as excellent and influential. Worse, these practices punish risk taking and innovation in a time when new approaches are most needed to solve the biggest challenges facing society. I am grateful for the leadership shown by Nielsen and Casadevall and for the opportunity to continue to build on this new tradition of science in pursuit of excellence through a positive research culture.
